# Locally delivered 1% metformin gel improves periodontal parameters: A systematic review and meta-analysis

**DOI:** 10.1007/s44445-025-00045-8

**Published:** 2025-08-20

**Authors:** Kata Sára Haba, Bulcsú Bencze, Gábor Kammerhofer, Márta Ujpál, Dorottya Bányai, Péter Hermann, Sarolta Antal, Klaudia Lipták, Laura Lipták, Zoltán Géczi, Tamás Hegedüs, Ádám Végh, Dóra Haluszka, Dániel Végh, Hamdan Alghamdi

**Affiliations:** 1 Department of Prosthodontics, Semmelweis University, Szentkirályi Street 47, 1088 Budapest, Hungary; 2 Department of Paediatric Dentistry and Orthodontics, Semmelweis University, Szentkirályi Street 47, 1088 Budapest, Hungary; 3 Department of Oro-Maxillofacial Surgery and Stomatology, Semmelweis University, Mária Street 52, 1088 Budapest, Hungary; 4 Institute of Biophysics and Radiational Biology, Semmelweis University, Tűzoltó Str. 37-47, 1094 Budapest, Hungary; 5 Diabetes Dental Research Team, Semmelweis University, Szentkirályi Street 47, 1088 Budapest, Hungary; 6 Periodontics and Dental Implants, College of Dentistry, King Saud University, 2454, 11451 Riyadh, Saudi Arabia

**Keywords:** Metformin, Periodontitis, Attachment loss, Periodontal pocket depth

## Abstract

**Purpose:**

Approximately 19% of the global adult population is affected by periodontal diseases. Healing is a protracted process that is significantly influenced by the patient's motivation, proficiency, and cooperation. The prevalence of periodontal diseases in patients with diabetes mellitus is extremely high, and the relationship is bidirectional. In previous investigations, metformin (MF), a biguanide antidiabetic medicine, has demonstrated promising results when administered locally to periodontal defects. The study aimed to systematically assess available literature that evaluate the effectiveness of 1% metformin gel in the treatment of periodontal disease. We attempted to include clinical trials on patients with periodontitis treated with local administration of 1% metformin gel, compared to a placebo.

**Methods:**

Using identical MeSH terms, we conducted a systematic search in three databases. Our analysis encompassed human studies that measured the periodontal pocket depth, the clinical attachment level, and the depth of the intra-bony defect. Meta-analyses were carried out using random effects model to compare the mean differences between the study group, which received 1% MF gel locally in addition to traditional scaling and root planing (SRP), and the control group, which received a placebo in addition to conventional treatment.

**Results:**

Following the selection procedure, six articles were selected for inclusion in our meta-analysis, out of the 246 articles identified through the systematic search. After three and six months of follow-up, meta-analyses revealed statistically significant differences between the control and study groups. The overall effect for PD was a 1.33 mm reduction (95% confidence interval (CI): -1.66; -1.01) after three months and 1.87 mm (-2.24; -1.39) after six months. The overall effect of CAL was 1.80 mm (-2.26; -1.34) after three months and 2.14 mm (-2.71; -1.58) after six months. The change in IBD after a six-month follow-up was 1.16 mm (-1.40; -0.92).

**Conclusion:**

The application of 1% MF gel enhances the healing process in periodontal diseases, when employed in conjunction with conventional therapy.

## Introduction

Metformin, a biguanide-type medication for diabetes mellitus (DM) is widely used in the treatment of type-2-diabetes mellitus (T2DM). In 1922, two Irish chemists, Emile Werner and James Bell, were the first to extract metformin from the medicinal goat's rue (Galega officinalis) in Dublin (Werner and Bell 1922). This drug has a beneficial effect on metabolic syndrome and insulin resistance by restoring the body's response to the hormone by reducing the amount of glucose released from the pancreas via gluconeogenesis by activating adenosine monophosphate kinase (AMPK). This inhibits vital enzymes in the process mentioned above and reduces their absorption from the gastrointestinal system, while not causing hypoglycemia (Winkler 2016). In 1993, H. Löe, Director of the American National Institute of Dental Research, pronounced periodontitis to be the sixth complication of DMs (Loe 1993). Periodontitis and diabetes are inextricably linked; both conditions can exacerbate insulin resistance (Borgnakke et al. 2013; Kalhan et al. 2024) and oxidative stress (Chen et al. 2023; Sun et al. 2024). Consequently, they are mutually and bi-directionally related and have a negative impact on one another (Borgnakke et al. 2015; Chiu et al. 2015). Metformin is effective in the reduction of inflammation and oxidative stress, as well as in the modulation of osteoblast apoptosis. It has been demonstrated in numerous studies to mitigate bone loss in periodontitis (Araujo et al. 2017; Jang et al. 2011). It is estimated that 10–15% of the global population is currently affected by this chronic oral inflammation, which primarily affects the tissues that support the teeth. This number is on the rise, with an estimated 1.5 billion individuals affected by 2050 (Fox 1992; Fox et al. 1994; Nascimento et al. 2024).Additionally, the combined presence of diabetes mellitus (DM) and periodontitis leads to an elevated mortality rate from diabetic nephropathy and ischaemic heart disease (Curry et al. 2018). These indicate that oral care for patients with T2DM who are periodontally affected is a comprehensive treatment.

Our objective is to address the clinical effectiveness of metformin treatment as an adjunct on periodontal parameters by conducting a systematic review and meta-analysis of the current literature.

## Materials and methods

### Search strategy

The present report was prepared in accordance with the Preferred Reporting Items for Systematic Reviews and Meta-analyses (PRISMA) (Dmytrenko et al. 2024), and the recommendations of Cochrane's Handbook. A protocol for the International Prospective Register of Systematic Reviews (PROSPERO) database was also obtained (ID: CRD42024542200). We conducted a systematic search in the available literature to address the following specific question: Is metformin effective in the treatment of periodontal disease when administered locally as adjunctive to non-surgical therapy?

The following PICO framework was used to answer this topic:

- The participants (P) were patients with periodontitis.
- The intervention (I) was metformin gel in 1% concentration, delivered locally.
- The comparators (C) were placebo materials.
- The outcomes (O) were objectively measurable periodontal parameters, like clinical attachment levels and periodontal pocket depth.

### Data sources & search strategy

We conducted our literature search electronically using three databases without restrictions based on publication date or language: MEDLINE (via PubMed), Embase, and CENTRAL (The Cochrane Central Register for Clinical Trials) up to 1 June 2023. We subsequently updated the search on January 6, 2025. The following search terms and protocols were employed: (metformin OR MF) AND (periodontitis OR probing depth OR clinical attachment OR regeneration). We used identical keywords and terms for each database.

### Eligibility criteria

The following inclusion criteria of studies were the following: randomized controlled trials, prospective studies, retrospective studies, or case series, that intended to measure periodontal pocket depth (PPD), clinical attachment level (CAL), and radiographic bone-level assessment at baseline, and after three and/or six months.

### Study selection & data extraction

The following were the exclusion criteria: review articles, animal studies, case reports, conference abstracts, full text unavailable, or written in a language other than English. Any duplicates in Titles and Abstracts were screened and removed by two independent reviewers. Then, same evaluators were verified the eligibility of full-texts. If necessary, a third independent reviewer was employed to resolve any disagreements. A standardized data extraction form was employed to collect and classify data from full-text.

### Risk of bias

Risk of bias of the included studies were assessed by two independent reviewers based on the RoB-2 risk of bias tool.

### Data synthesis and analysis

A random-effects model was employed to aggregate effect sizes, as we anticipated significant heterogeneity between studies. The effect magnitude was measured using the mean difference (MD) with 95% confidence intervals (CI) because the outcomes under investigation were continuous. Sample sizes, means, and the corresponding standard deviations (SD) were obtained from each study. The experimental group's values were subtracted from those of the control group. The results were deemed statistically significant regardless of whether the aggregated CI omitted the null value. Forest sites were utilized to illustrate the findings of the meta-analysis. The Higgins and Thompson I2 statistics (Higgins and Thompson 2002) were used to characterize the heterogeneity in between the studies. Basic meta-analysis calculations and visualizations were conducted using the meta package (Schwarzer 2023, v6.2.1) in R (R Core Team 2023, v4.3.0).

## Results

### Study selection

**Fig. 1:**
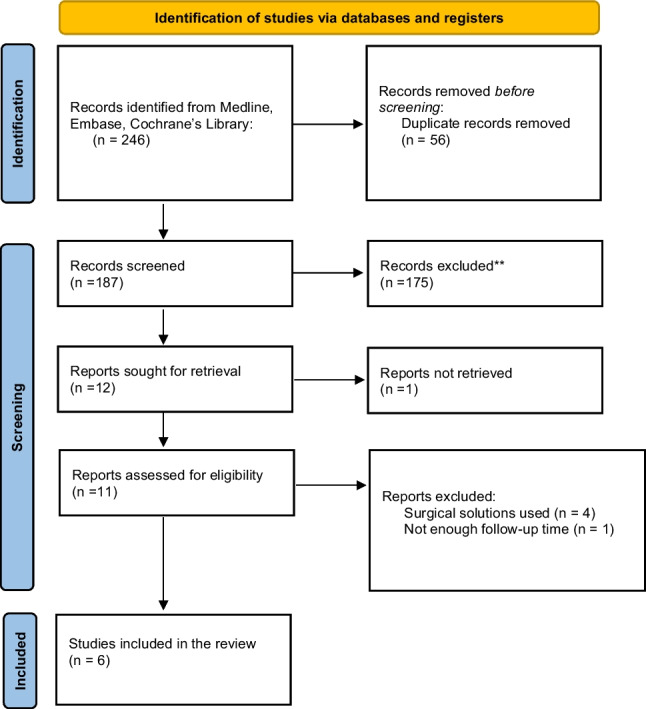
PRISMA Flowchart

The search strategy described above yielded a total of 246 articles. 187 documents were included in the selection phase after duplicates were removed. In the title-abstract selection phase, we selected 12 articles and conducted a search for full-text versions, provided that they were eligible. A total of 6 publications were incorporated into our meta-analysis after a meticulous selection process based on our pre-established inclusion and exclusion criteria (Fig. 1).

### Study characteristics

Articles included in our study were published between 2013 and 2018. all clinical trials were performed by same research group, using same population, from India. The studies included a total number of 246 patients, with a mean age of 36 (Table 1.).

**Table 1:** Basic characteristics of the included studies

First Author	Year	Country	No. of Patients	Conclusion
Pradeep et al. (2013)	2013	India	17	The 1% Metformin gel with scaling and root planing provided the best improvement in radiological and clinical parameters
Pradeep et al. (2016)	2016	India	60	Great reduction in periodontal pocket depth and increased gain in clinical attachment level
Pradeep et al. (2017)	2017	India	64	Locally delivered 1% Metformin gel stimulated a significant improvement in periodontal pocket depth and clinical attachment level
Pankaj et al. (2018)	2018	India	60	1% Metformin gel is an effective mode of treatment
Rao et al. (2013)	2013	India	45	The 1% MF gel, combined with scaling and root planing, improved both clinical and radiological parameters
Kurian et al. (2018)	2017	India	90	Local delivery of 1% MF gel significantly improves PPD reduction, CAL gain, and bone regeneration

### Results of synthesis

A meta-analysis was conducted to demonstrate the disparities between the experimental and control groups in terms of the depth of periodontal pocket, clinical attachment level, and the depth of intra-bony defect at 3 and 6 months, respectively. The measured results were statistically significant in all six analyses, as the confidence intervals of the data were significantly larger than the null-effect line.

### Risk of bias assessment

**Fig. 2:**
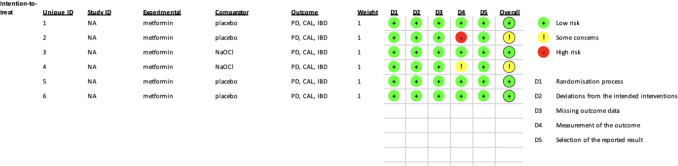
Risk of bias assessment of the included studies based on the RoB-2 tool

The RoB-2 risk of bias tool assigned a low overall rating to 3 of the included studies, while two received"some concerns"rating (Fig. 2). This tool is part of the Cochrane family of bias analyses, which evaluates randomized controlled trials using five domains. The first domain (D1) evaluates the randomization process; the second (D2) cares about possible deviations from the intended interventions; the third (D3) evaluates possible missing data; the fourth one (D4) helps measure the outcomes, and the fifth one (D5) helps to evaluate the selection of the reported results (Methods and RoB-2 tool (Cochrane Methods, n.d.) [Available from, https://www.riskofbias.info/welcome/rob-2-0-tool/current-version-of-rob-2.).

### Changes in periodontal pocket depth

**Fig. 3:**
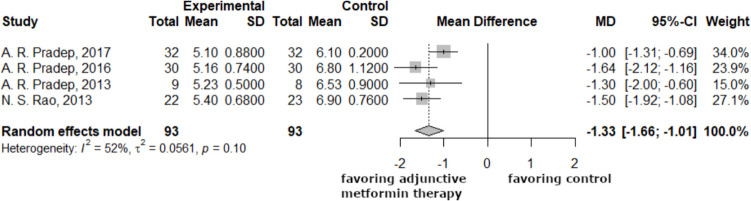
The average difference in periodontal pocket depth reduction was −1.33 mm (−1.66; 1.01) at the 3-month control. Negative mean difference indicates reduction in the periodontal pocket depth

**Fig. 4:**
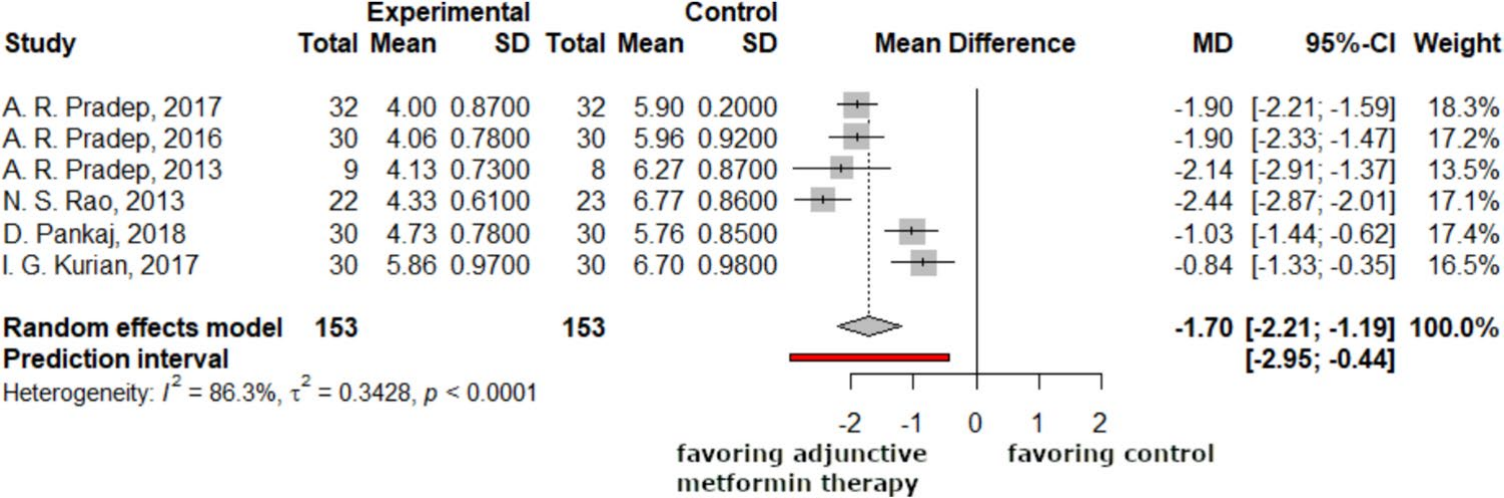
The average difference in periodontal pocket depth reduction was −1.70 mm (−2.21; −1.19) at the 6-months control. Negative mean difference indicates reduction in the periodontal pocket depth

The following forest plots (Figs. 3, 4) showed the differences between the 3-month and 6-month periodontal pocket depth data. After three and six months, the research revealed a statistically significant difference between the mean differences of the experimental and control groups'periodontal pocket depths in mm. The average depth of the periodontal pocket dropped by 1.33 mm after three months, with a 95% CI of −1.66 to −1.01. This number increased to 1.70 mm (−2.21; −1.19) after six months.

### Changes in clinical attachment levels

**Fig. 5:**
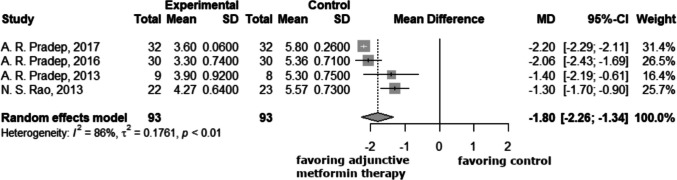
At the 3-month follow-up, the clinical attachment levels in the study group were on average 1.8 mm more reduced compared to the control group. Negative mean difference indicates reduction in the clinical attachment loss

**Fig. 6:**
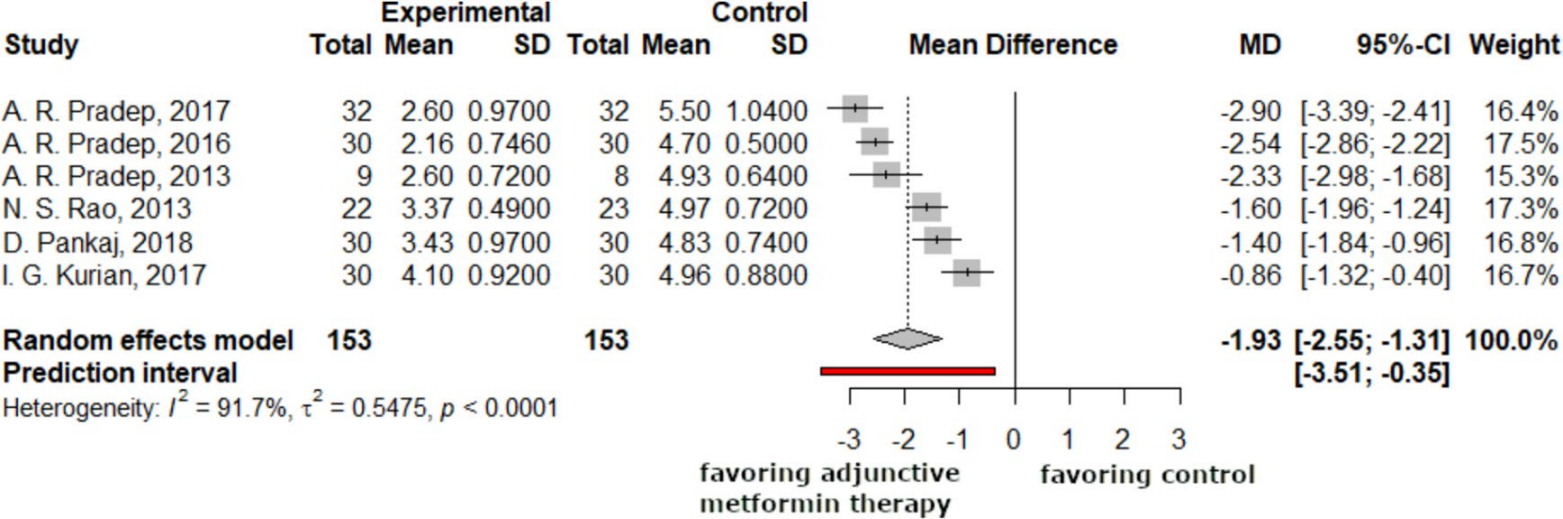
At the 6-month follow-up, the clinical attachment levels in the study group were on average 1.93 mm (2.56; 1.31) more reduced compared to the control group. Negative mean difference indicates reduction in the clinical attachment loss

The subsequent forest plots illustrated the variations in clinical attachment levels between the three-month and six-month time periods. The clinical attachment level was also significantly reduced in the study group at 3 and 6 months compared to the control group, with values of −1.80 (−2.26; −1.34) and −2.14 (−2.71; −1.58), respectively (Figs. 5, 6).

### Changes in intra-bony defect depth

**Fig. 7:**
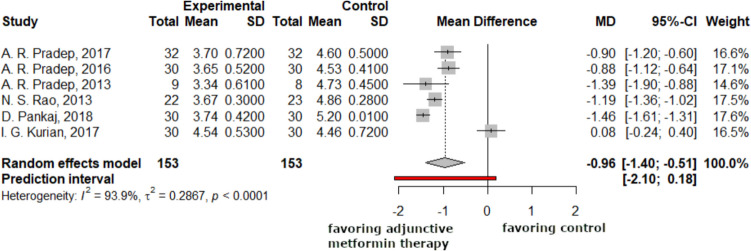
Based on the data available, the intra-bony defect depth in the study group was on average 1.16 mm more reduced than in the control group. Negative mean difference indicates reduction in the intra-bony defect depth

The following forest plots showed the changes in the 6-month intra-bony defect depth data, with statistically significant differences of −0.96 (−1.40; −0.51) (Fig. 7).

## Discussion

We performed a systematic literature review and meta-analysis to investigate the efficacy of locally applied 1% metformin gel in the treatment of periodontal diseases.

According to the Clinical Practice Guidelines established by the European Federation of Periodontology, the majority of periodontal diseases are managed following these guidelines (Sanz et al. 2020). The protocol includes recommendations that simultaneously address behavioural changes in the patient’s oral health management, options for the removal of supragingival biofilm and other risk factors for gingivitis, supra- and subgingival instrumental interventions, and periodontal surgical options. The patient's motivation and cooperation are crucial during the lengthy recuperating phase. A number of clinical studies are currently investigating agents that can assist both the patient and the doctor in accelerating and enhancing the rate of recovery. The efficacy of topically applied 1% metformin gel in comparison to conventional treatment was evaluated in the articles included in our review based on three critical parameters: PPD, CAL, and IBD. The three parameters were recalled at 3 and 6 months post-treatment. In patients who received 1% metformin gel in addition to conventional therapy, the meta-analysis of the data demonstrated that periodontal defects healed significantly more effectively at 3-month and 6-month follow-up.

Metformin is a first-line medication in T2DM, used systemically to help normalize blood glucose levels in patients with T2DM and impaired fasting glycemia (IFG) (Pernicova and Korbonits 2014). These patients are characterized by a lack of compliance, a poorer general status, higher chances of developing periodontal and peri-implant diseases, and thus a much higher prevalence of other co-morbidities (Bencze et al. 2024; Feldman et al. 2017; Madonna et al. 2017; Pecoits-Filho et al. 2016; Wang and Lo 2018; Ziebolz et al. 2018). One of the complications of T2DM is cancerous lesions in the head and neck region. Their incidence is increasing dramatically worldwide and in Hungary (Bosetti et al. 2020; Vegh et al. 2022). Previous studies have already shown that metformin, when administered systemically, reduces both the incidence and aggressiveness of not only head and neck cancer by almost 40% (Curry et al. 2018), but cancers in general. (O'Connor et al. 2024).

Periodontitis progression and tooth loss are closely linked to periodontal pocket depths, particularly when pockets exceed 5 mm in depth, rendering them unmaintainable by patients and fostering the expansion of the periodontopathogen microbiota (Antezack et al. 2023; Donos 2018; Greenstein et al. 1986). Porphyromonas gingivalis and Fusobacterium nucleatum are among the bacteria that manifest when the oral flora is unbalanced, resulting in the development of inflammations such as gingivitis or periodontitis. (Shaw et al. 2016). Non-surgical therapy seems to be effective in reducing periodontal pocket depths, and the effect of this therapy can be further enhanced by the additional of T2DM medication. This is particularly crucial for patients with comorbidities that are predisposed to the development of periodontitis, such as DM (Corbella et al. 2024; Hsu et al. 2019; Végh et al. 2023; Xu et al. 2024).

It may be necessary to alleviate postoperative distress and assist patients in their recovery following invasive periodontal surgeries. This can be achieved through the application of topical therapy. In this regard, metformin offers numerous benefits including its cost-effective, does not necessitate any specialized training, and can be employed by general dentists (Tseng 2022).

Our study demonstrated that the reduction can be reduced by 1.87 mm by incorporating topical metformin gel, while this number was 1.20 mm with conventional therapy, in accordance with previous research in the 6-month follow-up (Werner et al. 2024). These effects may be explained by the pleiotropic host-modulation and anti-inflammatory effects of metformin (Balta et al. 2021; Carvalho et al. 2024; Dmytrenko et al. 2024; Lindhe and Romandini 2024).

The clinical attachment is essential for the objective evaluation of the effects of periodontal therapy. Clinical attachment gains are estimated to be between 0.4 and 0.6 mm following non-surgical therapy (Gjermo 2005). Nevertheless, our investigation determined that the incorporation of topical metformin gel can result in gains of up to 1.7 mm, a substantial improvement over conventional therapy (Chang et al. 2019; Majzoub et al. 2021). Further research is required to investigate the impact of metformin gel on clinical attachment. Therefore, it has a significant effect on attachment gain, which can be more effectively employed in the clinical environment. The profundity of the intra-bony defect decreases as a result of the recovery of alveolar bone following the elimination of inflammation. In contrast to the 1.4 mm average defect depth reduction reported in other studies in the literature, our study discovered that the addition of metformin gel can result in an additional 1.1 mm of depth reduction (Harrington 2008; Kocher et al. 2018; Mehta et al. 2024; Wei et al. 2024; Xiao et al. 2017).

In addition to periodontitis, the healing time of oral wounds from other causes is significantly longer in DM patients. In addition to conventional treatments, it may be necessary to implement additional topical therapies. Different chemical agents, including chlorhexidine solution, hyaluronic acid, locally applied antibiotics, and topical oxygen-based therapy, could be applied locally in addition to metformin gel (Bhati et al. 2022; Greenstein et al. 1986; Herrera et al. 2020; Leventis et al. 2024). There are other frequently used adjuncts, such as laser treatments and locally administered antibiotics. According to the current literature, local antibiotics can improve periodontal parameters, and similarly, although results are still variable and technique-sensitive, laser-assisted periodontal therapy has shown promise in lowering periodontal inflammation and accelerating tissue repair (Mehravani et al. 2024; Theodoro et al. 2021). To determine the relative effectiveness and long-term advantages of metformin gel in comparison to other adjunctive therapies, more high-quality randomized controlled trials are required.

### Strengths and limitations

The articles included in our study are all recent and the studies are well-described, with a methodology that is straightforward to follow. These are the study’s strengths. The incidence rate of DM is higher in India than the global average (Pradeepa and Mohan 2021). Also, in rural areas access to health care is limited, that’s why it is valuable data. Our study, based on a pre-registered protocol, is the first to use statistical methods to examine the available articles in a meta-analysis. A limitation, however, is the limited number of articles available, often from the same author, originated from the same geographic region. Our results based on these show a high degree of heterogeneity and low confidence in our results. This could be likely explained by the differences in baseline CAL, IBD and PD values that suggest varying initial disease severity, which affects healing potential. Certain studies show small sample sizes, which reduce the stability of estimates. The interventions may differ in techniques and operator skills regarding root planing, and this can significantly affect outcomes. Although subgroup analysis is an efficient tool to investigate the abovementioned differences, it was not performed due to the limited number of studies included and their small sample sizes, that would have led to underpowered comparisons. These factors limit the generalizability of the findings to broader or more diverse populations. There is also a risk of overlapping patient populations across studies, particularly if they were conducted in the same clinical settings over similar timeframes, which could compromise the independence of the data and inflate sample sizes. These factors underscore the need for further validation through independent studies conducted in varied settings.

### Implications for future research

In order to conduct a more comprehensive analysis of the efficacy of metformin gel, additional studies with larger populations are required. It is also imperative to take into account anamnestic data that may impact bone regeneration, such as DM. An extended follow-up period would be beneficial for evaluating the long-term effects. It would also be prudent to compare the agent with other substances. The efficacy of metformin in periodontitis classified according to various severity categories should be examined.

## Conclusion

### Clinical relevance

Within the limitation of this study, we have found that local application of 1% metformin gel to periodontitis improves clinical outcomes.

### Implications for research

Further trials in larger populations would be needed for clinical application, but the agent showed promising results when used as an adjunct to conventional therapy.

## Data Availability

The datasets used and/or analyzed in the current study are available from the corresponding author upon reasonable request.
